# Two-Stage TMLE to reduce bias and improve efficiency in cluster randomized trials

**DOI:** 10.1093/biostatistics/kxab043

**Published:** 2021-12-23

**Authors:** Laura B Balzer, Mark van der Laan, James Ayieko, Moses Kamya, Gabriel Chamie, Joshua Schwab, Diane V Havlir, Maya L Petersen

**Affiliations:** Department of Biostatistics & Epidemiology, University of Massachusetts Amherst, 715 North Pleasant St, Amherst, MA, USA; Division of Biostatistics, University of California Berkeley, 2121 Berkeley Way, Berkeley, CA, USA; Center for Microbiology Research, Kenya Medical Research Institute, P.O. BOX 54840 00200 Off Raila Odinga Way, Nairobi, Kenya; Department of Medicine, Makerere University and the Infectious Diseases Research Collaboration, P.O Box 7475, Kampala, Uganda; Department of Medicine, University of California San Francisco, 995 Potrero Ave, San Francisco, CA, USA; Division of Biostatistics, University of California Berkeley, 2121 Berkeley Way, Berkeley, CA, USA; Department of Medicine, University of California San Francisco, 995 Potrero Ave, San Francisco, CA, USA; Division of Biostatistics, University of California Berkeley, 2121 Berkeley Way, Berkeley, CA, USA

**Keywords:** Clustered data, Cluster randomized trials, Covariate adjustment, Data-adaptive, Double robust, Group randomized trials, Missing data, Multi-level models, Super Learner, TMLE

## Abstract

Cluster randomized trials (CRTs) randomly assign an intervention to groups of individuals (e.g., clinics or communities) and measure outcomes on individuals in those groups. While offering many advantages, this experimental design introduces challenges that are only partially addressed by existing analytic approaches. First, outcomes are often missing for some individuals within clusters. Failing to appropriately adjust for differential outcome measurement can result in biased estimates and inference. Second, CRTs often randomize limited numbers of clusters, resulting in chance imbalances on baseline outcome predictors between arms. Failing to adaptively adjust for these imbalances and other predictive covariates can result in efficiency losses. To address these methodological gaps, we propose and evaluate a novel two-stage targeted minimum loss-based estimator to adjust for baseline covariates in a manner that optimizes precision, after controlling for baseline and postbaseline causes of missing outcomes. Finite sample simulations illustrate that our approach can nearly eliminate bias due to differential outcome measurement, while existing CRT estimators yield misleading results and inferences. Application to real data from the SEARCH community randomized trial demonstrates the gains in efficiency afforded through adaptive adjustment for baseline covariates, after controlling for missingness on individual-level outcomes.

## 1. Introduction

In many trials, treatments are randomly allocated to groups of individuals, such as hospitals, schools, or communities, and outcomes are measured on individuals in those groups. These studies are known as group or cluster randomized trials (CRTs). They are implemented when the treatment is naturally delivered to the group or when substantial dependence between individuals within groups is expected (Hayes and Moulton, 2009; Crespi, 2016; Turner *and others,* 2017a,b; Murray *and others,* 2020). CRTs are rapidly increasing in popularity; a recent review found a 280-fold increase in their use from 1995 to 2015 (Murray *and others,* 2018). Nonetheless, despite extensive research dedicated to their design and conduct, this review also concluded only half of CRTs were analyzed appropriately. CRTs can provide the gold-standard evidence of causality, but they face several methodological challenges.

First, missing participant outcomes occur in over 90% of CRTs (Fiero *and others,* 2016). When participants with missing outcomes differ meaningfully from those with measured outcomes, complete-case analyses yield biased estimates (Rubin, 1976; Robins *and others,* 1995). This potential for bias is exacerbated when, as commonly occurs, the cluster randomized intervention, itself, influences outcome measurement. Suppose, for example, that the cluster-level intervention increases care engagement, which in turn improves both participants’ outcomes and their chances of having that outcome measured (Figure S1 of the Supplementary material available at *Biostatistics* online). Here, an unadjusted comparison of outcomes between arms can overestimate or underestimate the treatment effect. Even if key determinants of missingness, such as care engagement, are measured, standard analytic approaches to CRTs will fail to control for this bias, because care engagement simultaneously mediates the treatment–outcome relationship and confounds the missingness–outcome relationship (Robins, 1986; Robins and others, 1995; Robins and Hernán, 2009).

Second, CRTs often randomize limited numbers of groups; a review found a median of 33 clusters randomized (Selvaraj and Prasad, 2013). Even when some form of restricted randomization (e.g., pair-matching) is used, CRTs with few clusters are likely to suffer from chance imbalances between treatment arms on baseline determinants of the outcome. Adjustment for these covariates and others predictive of the outcome (hereafter called “covariate imbalance”) can increase statistical power (e.g., Fisher, 1932; Gail *and others,* 1996; van der Laan and Rose, 2011; Hayes and Moulton, 2009; Colantuoni and Rosenblum, 2015; Turner *and others,* 2017b; Murray *and others,* 2018, 2020). The adjustment approach is often with an outcome regression, characterizing the expected outcome given the treatment assignment and covariates. This regression must be *a priori*-specified to avoid inflating Type-I error rates. Additionally to avoid over-fitting, a limited number of adjustment variables must be selected from a typically large set of candidates, risking forced adjustment for variables that prove useless for, or even detrimental to, precision (Stephens *and others,* 2013; Balzer *and others,* 2016; Kahan *and others,* 2016). Thus, we wish to define a fully prespecified procedure for CRT analysis that optimizes statistical power through data-adaptive adjustment of baseline covariates, while rigorously preserving Type-I error control.

In this article, we propose and evaluate a novel estimator that addresses the dual challenges of bias due to missing outcomes and imprecision due to few randomized units in CRTs. Our approach uses targeted minimum loss-based estimation (TMLE; van der Laan and Rose, 2011) in two stages: first at the individual-level to adjust for differential measurement of individual-level outcomes and second at the cluster-level to improve efficiency when estimating the intervention effect. Therefore, we refer to our estimator as “Two-Stage TMLE.” To the best of our knowledge, Two-Stage TMLE is the first semiparametric efficient estimator that adaptively adjusts for both individual-level missingness and for covariate imbalance in CRTs. It can be applied to estimate a range of causal parameters and under a range of CRT study designs, including differing randomization schemes (e.g., pair-matched or not) and approaches to participant follow-up within clusters (e.g., cross-sectional sampling or longitudinal follow-up).

## 2. Brief review of CRT Methods and overview of the motivating example

We provide an overview of existing CRT methods in Table 1 and refer the reader to Benitez *and others* (2021) for a detailed review. A simple two-stage approach to account for the dependence of participants within clusters is to aggregate the individual-level data to the cluster-level and then implement an effect estimator appropriate for independent data, such as a $t$-test. Use of an unadjusted effect estimator in the second stage avoids modeling assumptions and the risk of over-fitting, but by ignoring covariate information is inefficient (e.g.,Fisher, 1932; Gail *and others,* 1996; van der Laan and Rose, 2011; Hayes and Moulton, 2009; Colantuoni and Rosenblum, 2015; Turner *and others,* 2017b; Murray *and others,* 2018, 2020).

**Table 1.** Description of CRT effect estimators as commonly implemented when outcomes are completely measured

Unadjusted	Compare cluster-level outcomes by treatment arm; commonly implemented as a $t$-test.
CARE	At the cluster-level, compare observed outcomes with those predicted from a regression of the individual-level outcome on individual- and cluster-level covariates, but not the cluster-level treatment (Gail *and others,* 1996; Hayes and Moulton, 2009).
Mixed models	Point estimate and inference based the treatment coefficient in a regression of the individual-level outcome on the cluster-level treatment and individual- and cluster-level covariates; use random effects to account for dependence of individuals within a cluster (Laird and Ware, 1982).
GEE	Point estimate and inference based the treatment coefficient in a regression of the individual-level outcome on the cluster-level treatment and individual- and cluster-level covariates; use a working correlation matrix to account for dependence of individuals within a cluster (Liang and Zeger, 1986).
Augmented-GEE	Modification to GEE for the marginal effect (i.e., GEE with only regression coefficients for the intercept and cluster-level treatment) by including an additional augmentation term for the outcome regression (i.e., the conditional expectation of the outcome given covariates and treatment) (Stephens *and others,* 2012, 2013, 2014).
Hierarchical TMLE	Modification of TMLE for cluster-based exposures; initial predictions of the outcome regression and propensity score (i.e., conditional probability of treatment given the covariates) are made by adaptively selecting between individual- or cluster-level specifications (Balzer *and others,* 2019; Benitez *and others,* 2021; Supplementary material available at *Biostatistics* online).

CARE: Covariate adjusted residuals estimator

GEE: Generalized estimating equations

TMLE: Targeted minimum loss-based estimation

In contrast, mixed models and generalized estimating equations (GEE) typically adjust for a number of baseline individual- and cluster-level covariates, providing an opportunity to improve precision of effect estimates (Laird and Ware, 1982; Liang and Zeger, 1986). However, neither address the need for a prespecified approach to select the adjustment variables that optimize efficiency, while preserving valid statistical inference. Further, both are susceptible to allowing the estimator choice define the effect measure that is estimated (e.g., GEE with a logistic link yields estimates of the conditional odds ratio) (Hubbard *and others,* 2010).

To the best of our knowledge, only three methods generally allow for estimation of marginal effects in CRTs, while adjusting for individual- and cluster-level covariates. First, in the covariate adjusted residuals estimator (CARE), cluster-level outcomes are compared with those predicted from an individual-level regression of the outcome on individual- and cluster-level covariates, but not the cluster-level treatment (Gail *and others,* 1996; Hayes and Moulton, 2009). Second, augmented-GEE extends GEE for the marginal effect by including an “augmentation” term, inspired by the efficient influence function (Stephens *and others,* 2012, 2013, 2014). Finally, hierarchical TMLE extends TMLE for estimation of marginal effects with cluster-based exposures (Balzer *and others,* 2019; Benitez *and others,* 2021; overview in Supplementary material available at *Biostatistics* online).

With regards to missingness, an unadjusted effect estimator requires the strongest identification assumption: there are no common causes of missingness and outcomes (i.e., the missing-completely-at-random, or MCAR, assumption holds) (Rubin, 1976). The other methods rely on a weaker identification assumption; essentially, that the outcome distributions among persons for which the outcome is measured versus missing are exchangeable conditional on the treatment arm and some subset of measured covariates (Turner *and others,* 2017b; Hossain *and others,* 2017a,b).

Combining augmented-GEE with inverse probability weighting yields a double robust estimator (“DR-GEE”); it is nearly unbiased if either the outcome regression or the measurement mechanism (i.e., the conditional probability of outcome measurement given the treatment arm and covariates) is correctly specified (Prague *and others,* 2016). To the best of our knowledge, DR-GEE’s methodology and computing code have been limited to adjustment for baseline variables only. Hierarchical TMLE also offers the potential for integrated precision gains and double robust adjustment. However, these extensions remain to be fully studied. Here, we, instead, develop and evaluate Two-Stage TMLE to (i) control for potentially differential missingness in each cluster separately and (ii) adaptively adjust for covariate imbalance to improve efficiency when estimating the intervention effect. Before doing so, we present our motivating example.

The SEARCH Study was a pair-matched, pragmatic CRT of 32 communities each with 10 000 persons in rural Kenya and Uganda (ClinicalTrials.gov: NCT01864603) (Havlir *and others,* 2019). SEARCH was designed to evaluate the population-level effects of annual multi-disease testing and universal, patient-centered treatment for persons with HIV (intervention) versus baseline multi-disease testing and country-guided treatment (active control) on a range of outcomes including incident HIV, viral suppression among persons with HIV, hypertension control, and incident tuberculosis (TB).

As with the vast majority of CRTs, outcomes were not measured among all SEARCH participants and the MCAR assumption was unreasonable for many endpoints. Additionally, despite matching prior to randomization (Balzer *and others,* 2015), covariate imbalance was expected; however, it was unclear *a priori* which covariates to include in the adjustment set for optimal gains in efficiency. To reduce bias from missingness on individual-level outcomes and to maximize precision during effect estimation in the SEARCH Study, we developed Two-Stage TMLE.

## 3. Two-Stage TMLE

In many CRTs, outcomes are assessed through longitudinal follow-up of a closed cohort of participants. In the SEARCH Study, for example, the primary outcome was the 3-year cumulative incidence of HIV: the proportion of community residents ($\geq$15 years) who were HIV-uninfected at baseline and became infected with HIV over the 3-year study. To assess the treatment effect on such endpoints, a cohort of participants who are at risk of the outcome is defined in each cluster. For each participant, let $W$ denote their baseline covariates, $M$ be their postintervention covariates, $\Delta$ be an indicator of outcome measurement, and $Y$ be the outcome of interest. The outcome $Y$ is only observed when $\Delta=1$. We also observe cluster-level covariates $E^c$ and the randomly assigned cluster-level intervention $A^c$. Throughout, superscript $c$ will be used to distinguish cluster-level variables from individual-level variables.

We denote the observed data structure for a participant as $O =\left (E^c, W, A^c, M, \Delta, \Delta Y \right)$. In the SEARCH Study, for example, $E^c$ included baseline HIV prevalence and male circumcision coverage; $W$ included age, sex, marital status, occupation, education, and mobility; $A^c$ was a community-level indicator of randomization to the intervention; $M$ was interim HIV testing; $\Delta$ was an indicator of HIV testing at year 3, and $Y$ was an indicator of having a confirmed HIV-positive diagnosis at year 3 testing.

**Fig. 1.**
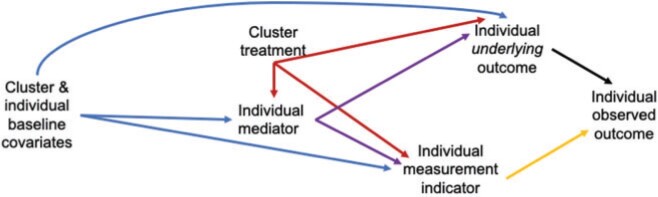
Simplified causal graph to illustrate the challenges of a single stage approach to identifying effects defined by interventions on both the cluster-level treatment and individual-level measurement mechanism.

**Fig. 2.**
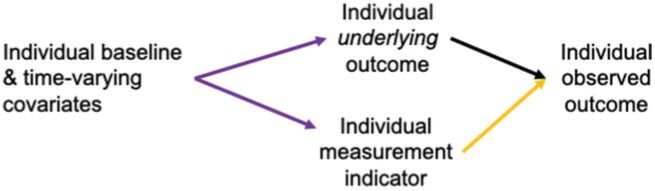
Simplified causal graph to illustrate how stratifying on cluster in Stage 1 simplifies identification and estimation of the Stage 1 statistical parameter $Y^c$, corresponding to hypothetical intervention to ensure complete outcome measurement. The Stage 1 estimates $\hat{Y}^c$ are used to evaluate the intervention effect in Stage 2.

Recall our goal is to simultaneously control for differential missingness on individual-level outcomes, while estimating the effect of the cluster-level intervention with optimal precision. To do so, we consider an individual-level counterfactual outcome $Y(a^c, \delta)$, generated by hypothetical interventions on the cluster-level treatment (i.e., to “set” $A^c=a^c$) and the individual-level measurement mechanism (i.e., to “set” $\Delta=\delta$). Identification of a corresponding causal parameter (e.g., $\mathbb{E}[Y(a^c,1)]$) is complicated by clustering and the missing data equivalent to time-dependent confounding (Figure 1). Instead, in our novel Two-Stage approach, we separate control for missing outcomes (Stage 1) from evaluation of the intervention effect (Stage 2). Specifically, in Stage 1, we fully stratify on each cluster, vastly simplifying identifiability and estimation for the missing data problem (Figure 2). Then in Stage 2, we use the estimates from Stage 1 to evaluate the intervention effect. Our approach allows the missingness mechanism to vary by cluster, while avoiding specifying complex relationships between individual-level ($W, M, \Delta$, $\Delta Y$) and cluster-level variables ($E^c, A^c)$. As detailed below, our Two-Stage approach is also applicable to setting with more complicated missingness mechanisms, such as right-censoring with survival-type endpoints.

### 3.1. Stage 1: Identifying and estimating cluster-specific endpoints

For the purposes of controlling for differential outcome measurement, we consider each of the $N$ clusters separately in Stage 1. Since the cluster-level covariates and treatment $(E^c,A^c)$ are constant within each cluster, the Stage 1 observed data simplify to $O=(W,M,\Delta,\Delta Y)$ and the target causal parameter to $\mathbb{E}[Y(\delta=1)]$. Then if MCAR held in each cluster or, equivalently, $Y(1) \perp\!\!\!\perp \Delta$, this causal parameter could be identified as $\mathbb{E}(Y| \Delta=1)$ and consistently estimated as the empirical mean among those measured within each cluster: $\hat{\mathbb{E}}(Y | \Delta=1)$. We relax this missing data assumption by allowing measurement to depend on the participant’s baseline and time-varying characteristics $(W,M)$. Specifically, if $Y(1) \perp\!\!\!\perp \Delta \mid W, M$ and there is sufficient data support (i.e., the positivity assumption holds), our Stage 1 statistical estimand would be

(3.1) \begin{equation*} \label{HIV} Y^c \equiv \mathbb{E}\big[ \mathbb{E}(Y \big| \Delta=1,W,M)\big].\end{equation*}

Within each cluster separately, $Y^c$ could be estimated by a variety of algorithms, including inverse-weighting and G-computation (Horvitz and Thompson, 1952; Robins, 1986). We use TMLE for estimation of the cluster-specific endpoint $Y^c$, given its asymptotic properties and improved finite sample performance (e.g., van der Laan and Rose, 2011; Gruber and van der Laan, 2012). Briefly, TMLE combines estimates of the outcome regression $\mathbb{E}(Y| \Delta=1,W,M)$ with those of the measurement mechanism $\mathbb{P}(\Delta=1|W,M)$. In doing so, TMLE achieves a number of desirable properties, including double robustness: a consistent estimate is attained if either the outcome regression or the measurement mechanism is consistently estimated. If both are consistently estimated at reasonable rates, TMLE will be efficient. In practice, we recommend implementing TMLE using Super Learner, an ensemble machine learning algorithm (van der Laan *and others,* 2007). Step-by-step implementation for Eq. 3.1 is given in the Supplementary material available at *Biostatistics* online. Since we are fully stratifying on cluster, an individual-level TMLE would be implemented $N$ times to obtain $N$ cluster-specific estimates: $\hat{Y}^c = \frac{1}{S^c} \sum_{j=1}^{S^c} \hat{\mathbb{E}}^*(Y \big| \Delta=1,W_j, M_j)$, where $j$ indexes the $S^c$ participants in a given cluster and $\hat{\mathbb{E}}^*(Y| \Delta=1,W_j, M_j)$ denotes the targeted prediction of the individual-level outcome for participant $j$ in that cluster.

#### 3.1.1. Stage 1 with survival-type endpoints:

When assessing effects on time-to-event outcomes, participants are followed longitudinally until the occurrence of the event of interest or right-censoring. Examples of such endpoints in the SEARCH Study included the probability of treatment initiation and the cumulative risk of HIV-associated TB or death due to illness. Our framework also accommodates survival-type endpoints. Specifically, to account for right-censoring in Stage 1, we would identify a new cluster-specific endpoint $Y^c$ and estimate it using the Kaplan-Meier estimator when censoring is nondifferential or TMLE when censoring is differential (Petersen *and others,* 2014; Benkeser *and others,* 2019).

#### 3.1.2. Stage 1 with endpoints measured in a cross-sectional design:

Other endpoints may be assessed using a cross-sectional design, where participants are measured at a single timepoint at the conclusion of the trial. In these settings, we may have missingness on the characteristic defining the subpopulation of interest as well as the outcome of interest. Consider, for example, population-level HIV viral suppression, defined as the proportion of all HIV-infected persons whose plasma HIV RNA level is suppressed below some limit: $\mathbb{P}(Suppressed \mid HIV+)$. Both baseline and postbaseline factors impact HIV status and its measurement as well as viral suppression and its measurement. To handle missingness on both the outcome (viral suppression) and the conditioning set (HIV-positivity), we redefine the outcome as the joint probability of being HIV-infected and suppressing viral replication, divided by HIV prevalence: $\mathbb{P}(Suppressed, HIV+) \div \mathbb{P}(HIV+)$. Again our Two-Stage approach can accommodate this ratio-type endpoint. Specifically, we would identify estimate (with TMLE) the numerator and denominator separately, and then take their ratio to estimate a new cluster-specific endpoint $Y^c$ in Stage 1 (Balzer *and others,* 2020).

### 3.2. Stage 2: Estimation of the effect of the cluster-level intervention

Recall in Stage 1, we stratify on each cluster to identify and estimate a cluster-specific endpoint $Y^c$ that accounts for potentially differential measurement or censoring at the individual-level (e.g., 3.1). Then in Stage 2, our goal is to use those estimates to evaluate the intervention effect with maximum precision. Supplementary material available at *Biostatistics* online provides a detailed discussion of several Stage 2 causal effects, which are all easily identified due to randomization of the cluster-level treatment $A^c$ and prior control for missingess in Stage 1. For estimation of these effects in Stage 2, the observed data can be simplified to the cluster-level: $O^c = (E^c, W^c, A^c, \hat{Y}^c)$, where $E^c$ represents the baseline cluster-level covariates; $W^c$ denotes summary measures of the baseline individual-level covariates; $A^c$ is an indicator of randomization to the intervention arm, and $\hat{Y}^c$ is the estimated cluster-specific endpoint from Stage 1.

With these data, a simple estimator of the treatment effect is the average outcome among intervention clusters $\hat{\mathbb{E}}(\hat{Y}^c| A^c=1)$ contrasted with the average outcome among control clusters $\hat{\mathbb{E}}(\hat{Y}^c| A^c=0)$. Instead, to obtain a more efficient estimate of the intervention effect (e.g., Moore and van der Laan (2009); Rosenblum and van der Laan (2010)), we implement a cluster-level TMLE in Stage 2. Briefly, an initial estimator of the cluster-level outcome regression $\mathbb{E}(\hat{Y}^c | A^c, E^c, W^c)$ is updated based on an estimate of the cluster-level propensity score $\mathbb{P}(A^c=1| E^c, W^c)$ to achieve a targeted estimator $\mathbb{E}^*(\hat{Y}^c | A^c, E^c, W^c)$. Targeted estimates of the expected outcomes under the intervention $\mathbb{E}^*(\hat{Y}^c | A^c=1, E^c, W^c)$ and under the control $\mathbb{E}^*(\hat{Y}^c | A^c=0, E^c, W^c)$ are generated for all clusters, averaged across clusters, and contrasted. Step-by-step implementation of TMLE in Stage 2 is given in Supplementary material available at *Biostatistics* online.

Unfortunately, it is nearly impossible to *a priori*-specify the optimal estimators of the outcome regression $\mathbb{E}(\hat{Y}^c | A^c, E^c, W^c)$ or the propensity score $\mathbb{P}(A^c=1| E^c, W^c)$. Few clusters prohibit the use of Super Learner. To avoid over-fitting while flexibly selecting the adjustment variables that maximize precision, we recommend using *Adaptive Prespecification* in Stage 2 (Balzer *and others,* 2016). The procedure data-adaptively selects from a pre-specified set the candidate adjustment variables and, thus, the TMLE that maximize empirical efficiency. Briefly, we prespecify (i) candidate adjustment variables expected to be predictive of the outcome, (ii) a loss function corresponding to the squared influence curve for the TMLE of the target effect, and (iii) a sample-splitting scheme; leave-one-out cross-validation is recommended. The candidate adjustment variables then define the set of candidate estimators for the outcome regression and the propensity score based on “working” generalized linear models (GLMs). The procedure data-adaptively choses the combination of the outcome regression and propensity score GLMs (and thus the TMLE) with the lowest cross-validated variance estimate. Adaptive Prespecification is an extension of Collaborative-TMLE using a cross-validation selector to maximize precision in small trials.

In CRTs with few clusters, we recommend limiting the candidate GLMs to a single adjustment covariate. In CRTs with many clusters, we could also include candidate GLMs adjusting for multiple covariates and allow the procedure to data-adaptively determine the size of the adjustment set. Simulations mimicking the real data application (e.g., in sample size $N$ and expected within cluster dependence) can help inform these choices. Importantly, there is no guarantee that adjusting for more covariates will improve empirical efficiency. For some CRTs, none of the prespecified covariates will improve precision over the unadjusted estimator. In this setting, the procedure will select the unadjusted effect estimator. Altogether with Adaptive Prespecification, decisions about whether and how to adjust for optimal precision gains in Stage 2 are made with a rigorous procedure that does not compromise Type-I error.

### 3.3. Statistical inference for Two-Stage TMLE

Now we have a point estimate of the intervention effect and are ready to obtain statistical inference, which occurs at the cluster-level. Under the following conditions, detailed in Supplementary material available at *Biostatistics* online, Two-Stage TMLE $\hat{\psi}$ is an asymptotically linear estimator of the intervention effect $\psi$, meaning that $\hat{\psi}- \psi = \frac{1}{N}\sum_{i=1}^N {\rm IC}_i + R_N$, where ${\rm IC}_i$ is the cluster-level influence curve and $R_N= o_P(N^{-1/2})$ is the remainder term, going to zero in probability (van der Vaart, 1998):

(1) Stage 1 estimation of the cluster-level outcomes $\hat{Y}^c$ provides negligible contribution to $R_N$;
(2) Stage 2 estimators of the cluster-level outcome regression and the cluster-level propensity score satisfy the usual regularity conditions (e.g., Moore and van der Laan, 2009).

The second condition is automatically satisfied when Adaptive Prespecification is used to select among GLMs for the outcome regression $\mathbb{E}(\hat{Y}^c | A^c, E^c, W^c)$ and the known propensity score $\mathbb{P}(A^c=1 | E^c, W^c)$. However, to satisfy the first condition we need (i) the Stage 1 estimators of the individual-level outcome regression and the individual-level measurement mechanism to converge to their targets at fast enough rates, (ii) the within cluster dependence to be weak enough that the Central Limit Theorem applies in cluster size $S_i^c$, and (iii) the cluster size is large relative to the total number of clusters (i.e., $N/{\rm min}(S_i^c) \rightarrow 0$; Supplementary material available at *Biostatistics* online).

While these are asymptotic requirements, the Stage 1 conditions highlight the importance of having sufficiently sized clusters to support our missing data assumptions and to allow for flexible estimation of the individual-level outcome regression and measurement mechanism in Stage 1. Small cluster sizes may force us to rely on strong identifiability assumptions (e.g., MCAR within each cluster or only dependent on a single covariate) and strong estimation assumptions (e.g., the probability of being measured is accurately described by a main terms logistic regression). Such assumptions may or may not be reasonable in a given application. Larger cluster sizes, however, permit the use TMLE with Super Learner to flexibly adjust for the baseline and time-dependent covariates influencing outcomes and measurement in each cluster in Stage 1. TMLE is also double robust, providing a consistent estimate of the cluster-specific endpoint $Y^c$ if either the individual-level outcome regression or measurement mechanism is consistently estimated. Altogether, the requirements for valid statistical inference are not unique to our proposed approach; they apply to other two-stage estimators (e.g., a $t$-test on the mean outcome among those measured). In all cases, if the cluster-level endpoints $Y^c$ are estimated poorly in Stage 1, we are risk of biased point estimates and misleading conclusions in Stage 2. Simulations, below, explore the finite sample performance of Two-Stage TMLE under challenges commonly faced by CRTs: few clusters of modest size, correlated outcomes within clusters, and differential outcome measurement.

When the above conditions hold, the limit distribution of the standardized estimator is normal with mean 0 and variance given by the variance of its influence curve. For the treatment-specific mean $\psi(a^c)=\mathbb{E}[\mathbb{E}(Y^c|A^c=a^c, E^c,W^c)]$, for example, the influence curve for Two-Stage TMLE is approximated as

$$
\hat{IC}(a^c) = \frac{\mathbb{I}(A^c=a^c)}{\hat{\mathbb{P}}(A^c=a^c | E^c,W^c)} [\hat{Y}^c - \hat{\mathbb{E}}^*(\hat{Y}^c | A^c=a^c, E^c, W^c) ]
+ \hat{\mathbb{E}}^*(\hat{Y}^c | A^c=a^c, E^c, W^c) - \hat{\psi}(a^c).
$$

We obtain a variance estimate with the sample variance of the estimated influence curve divided by the number of independent units $N$. Then using the Student’s $t$-distribution with $N-2$ degrees of freedom as a finite sample approximation to the normal distribution (Hayes and Moulton, 2009), we can construct Wald-Type 95% confidence intervals and conduct hypothesis testing. Additionally, through the Delta Method, we can derive the influence curve and variance estimator for the intervention effect on any scale of interest. For the absolute effect $\psi(1) - \psi(0)$, the estimated influence curve for TMLE would be $\hat{IC}(1) - \hat{IC}(0)$. For the relative effect $\psi(1) \div \psi(0)$, we would apply the Delta method on the log-scale (Moore and van der Laan, 2009). This approach to statistical inference also applies when the treatment is randomized within matched pairs of clusters (Supplementary material available at *Biostatistics* online).

## 4. Simulation study

We examine the finite sample performance of our proposed estimator using simulations to incorporate common CRT challenges, such as few randomized clusters and differential missingness. Specifically, we focus on a setting with $N=30$ clusters and where within each cluster, the number of individual participants is sampled with equal probability from $\{100, 150, 200\}$. In these simulations, both baseline and postbaseline covariates impact measurement of individual-level outcomes. Additional simulations and computing code are given in the Supplementary material available at *Biostatistics* online.

### 4.1. Data generating process

For each cluster $i=\{1,\ldots, N\}$, we independently generate the cluster-specific data as follows. First, three cluster-level, latent variables are independently generated as $U1^c \sim Unif(-1,1)$, $U2^c \sim Unif(-1, 1)$, and $U3^c\sim Norm(0,1)$. Then, two individual-level covariates $(W1,W2)$ are drawn independently from normal distributions with cluster-specific means: $W1 \sim Norm(U1^c, 0.5)$ and $W2 \sim Norm(U2^c, 0.5)$. We set the observed cluster-level covariates $(E1^c, E2^ c)$ to be the empirical mean of their individual-level counterparts. The cluster-level intervention $A^c$ is randomly allocated within pairs of clusters matched on $U3^c$.

The individual-level mediator $M$ is generated as an indicator that $U_M\sim Unif(0,1)$ is less than the $logit^{-1}\{-1 + 2 A^c + W1 + W2 + 0.2(1-A^c)( E1^c + E2^c) + 0.25U3^c\}$. The underlying, individual-level outcome $Y$ is generated as an indicator that $U_Y\sim Unif(0,1)$ is less than $logit^{-1}(1 -2.5A^c + 4M + 0.5W1 + 0.5W2 + 0.2E1^c + 0.2 E2^c + 0.25U3^c)$. Finally, individual-level measurement is generated as an indicator that $U_\Delta \sim Unif(0,1)$ is less than $A^c logit^{-1}(3 -3M - 0.5W1 - 0.5W2) + (1-A^c) logit^{-1}(-2 +3M + 0.5W1 + 0.5W2)$. Thus, the measurement mechanism is highly differential by treatment arm with a mean of 70% measured in the intervention arm and 43% in the control arm. The observed outcomes $Y$ are set to missing for individuals with $\Delta=0$.

We also generate the counterfactual mediators and outcomes by setting the cluster-level treatment $A^c=a^c$ and preventing missingness (i.e., setting $\Delta=1$). The cluster-level, counterfactual outcome $Y^c(a^c)$ is the average of the individual-level, counterfactual outcomes within each cluster. We generate a population of 5000 clusters and calculate the true value of the treatment-specific, population means $\mathbb{E}[Y^c(a^c)]$ for $a^c=\{0,1\}$, their difference, and their ratio.

### 4.2. Estimators compared in the simulation study

We compare a variety of estimators commonly implemented in CRTs. We consider four complete-case approaches, in which the data are subset to exclude participants with missing outcomes (i.e., those with $\Delta=0$): an unadjusted estimator, CARE, mixed models, and GEE. We also implement two approaches which use data on all participants: DR-GEE and our Two-Stage TMLE.

For the unadjusted approach, we first aggregate the individual-level outcomes $Y$ to the cluster-level $\hat{Y}^c$ by taking the empirical mean among those measured (i.e., $\Delta=1$) and then contrast the average cluster-level outcomes $\hat{Y}^c$ by treatment arm $A^c$ with inference from the $t$-distribution. For CARE, we pool data across clusters and run logistic regression of the individual-level outcome $Y$ on the baseline covariates $(W1, W2, E1^c, E2^c)$; calculate the residuals by taking the difference between the cluster-level outcomes $\hat{Y}^c$ (the empirical mean among those measured) and those predicted from the previous regression, and finally use a $t$-test to compare the residuals by arm.

In mixed models and GEE, we again pool data across clusters and fit a log-linear regression of the individual-level outcome $Y$ on the cluster-level treatment $A^c$ and baseline covariates $(W1, W2, E1^c, E2^c)$. In DR-GEE, we also estimate the measurement mechanism with a pooled logistic regression of $\Delta$ on $(W1, W2, E1^c, E2^c, A^c)$ and the augmentation terms with arm-specific log-linear regressions of $Y$ on $(W1, W2, E1^c, E2^c)$. To account for within cluster dependence, we include a random cluster-specific intercept in mixed models and use an independent working correlation matrix in the GEEs. For mixed models, GEE, and DR-GEE, the default settings of $\texttt{lme4}$, $\texttt{geepack}$, and $\texttt{CRTgeeDR}$ packages are used for standard error estimation, respectively.

For Two-Stage TMLE, we first implement an individual-level TMLE within each cluster separately to estimate the cluster-specific endpoint $Y^ c \equiv \mathbb{E}\big[\mathbb{E}(Y \mid \Delta=1, W1, W2, M) \big]$. In these TMLEs, the outcome regression and the measurement mechanism are estimated using Super Learner to combine predictions from main terms logistic regression, generalized additive models, and the empirical mean. In Stage 2, we compare these cluster-specific estimates $\hat{Y}^c$ by treatment arm using a cluster-level TMLE with Adaptive Prespecification to select the optimal adjustment variables from $\{E1^c, E2^c, \emptyset \}$ and with inference via the estimated influence curve (Section 3.3).

### 4.3. Simulation results

The average coefficient of variation was 0.24 in the intervention arm and 0.17 in the control arm, reflecting expected levels of dependence between individual-level outcomes within clusters (Hayes and Moulton, 2009). The true values of the risk difference and risk ratio were $-$9.1% and 0.88, respectively. For both effects, Table 2 summarizes estimator performance when “breaking the matches” (i.e., ignoring the pair-matching scheme used for treatment randomization) and when preserving the matches. The exception is for DR-GEE, because to our knowledge, there does not yet exist an extension of DR-GEE for pair-matched CRTs.

**Table 2.** Over 500 simulated trials, the performance of CRT estimators when missingness depends on baseline and post-baseline variables. Results are shown when the target of inference is the risk difference (RD; top 3 rows), when the target is the risk ratio (RR; bottom 4 rows), when breaking the matches during analysis (left), and when preserving the matches during analysis (right)

	Breaking the matches						Keeping the matches					
	$\hat{pt}$	Bias	$\sigma$	$\hat{\sigma}$	CI	Power	$\hat{pt}$	Bias	$\sigma$	$\hat{\sigma}$	CI	Power
For the risk difference (true value RD = $-$9.1%)												
t-test	–32.0	–22.9	0.048	0.050	0.8	100.0	–32.0	–22.9	0.048	0.047	0.6	100.0
CARE	–21.8	–12.7	0.037	0.037	7.8	100.0	–19.0	–9.9	0.049	0.040	40.4	98.0
TMLE	–9.8	–0.7	0.038	0.046	98.8	52.8	–9.9	–0.8	0.037	0.043	96.6	57.4
For the risk ratio (true value RR = 0.88)												
Mixed	0.7	–0.2	0.049	0.069	7.0	100.0	0.7	–0.2	0.050	0.065	5.6	100.0
GEE	0.7	–0.2	0.049	0.056	4.8	100.0	0.7	–0.2	0.055	0.036	1.2	99.8
DR-GEE	0.7	–0.2	0.049	0.054	0.2	100.0						
TMLE	0.9	–0.0	0.051	0.063	98.4	52.6	0.9	–0.0	0.051	0.058	96.8	57.8

$\hat{pt}$ : average point estimate (in % for the RD).

Bias: average deviation in the point estimates versus true effect (in % for the RD).

$\sigma$ : standard deviation of the point estimates (on log-scale for RR).

$\hat{\sigma}$ : average standard error estimate (on log-scale for RR).

CI: proportion of 95% confidence intervals containing the true effect (in %).

Power: proportion of trials correctly rejecting the false null hypothesis (in %).

Focusing first on estimators of the risk difference (true value = $-$9.1%), we see that $t$-test, which does not adjust for covariates, is highly biased, as expected given the differential measurement process. On average, it grossly overestimates the effect by 22.9% and attains confidence interval coverage of $<$1%. By adjusting for covariates, CARE is less biased, but still overestimates the intervention effect by 12.7% when breaking the matches and by 9.9% when preserving the matches. The corresponding confidence interval coverages are much less than the nominal rate: 7.8% and 40.4%, respectively. In contrast, the bias of Two-Stage TMLE is low ($<$1%) and confidence interval coverage is good ($>$95%). Also as predicted by theory (Balzer *and others,* 2015), more power is achieved when preserving (57.4%) versus breaking the matches (52.8%).

Now focusing on estimators of the risk ratio (true value = 0.88), both mixed models and GEE overestimate the effect, preventing accurate inference. The confidence interval coverage is 5.6–7.0% for mixed models and 1.2–4.8% for GEE. These estimators are expected to be unbiased when there are only baseline causes of missingness and the outcome regressions are correctly specified. Here, there are postbaseline causes of missingness, which are simultaneously mediators of the treatment–outcome relationship. DR-GEE is expected to reduce bias due to missing outcomes by incorporating weights corresponding to the measurement mechanism, and this is seen when there are only baseline causes of missingness (Table S1 of the Supplementary material available at *Biostatistics* online). However, extensions of DR-GEE to handle postbaseline causes of missingness do not yet exist, and in the main simulations, DR-GEE attains $<$1% confidence interval coverage for the risk ratio (Table 2).

In contrast, Two-Stage TMLE is essentially unbiased and achieves good confidence interval coverage ($>$95%) for the risk ratio. Again, more power is achieved when keeping (57.8%) versus breaking the matches (52.6%). In further simulation studies given in the Supplementary material available at *Biostatistics* online, Two-Stage TMLE also performs well with fewer clusters and maintains nominal Type-I error control when there is no effect. Finally, including the mediator $M$ in the adjustment set of the existing methods does not improve their performance. (Table S2 of the Supplementary material available at *Biostatistics* online).

## 5. Application to the SEARCH Study

The results of the SEARCH Study have been previously published in Havlir *and others* (2019); here, we focus on the efficiency gains from adjusting for covariate imbalance in Stage 2, *after* adjusting for individual-level missingness in Stage 1. For select endpoints, we describe the estimator implementation and then compare point estimates and inference for the intervention effect when using TMLE with Adaptive Prespecification versus the unadjusted effect estimator in Stage 2.

As previously discussed, the primary outcome in the SEARCH Study was the 3-year cumulative HIV incidence, measured in each community through a cohort of residents who were aged 15+ years and HIV-uninfected at baseline. The pre-specified primary approach was Two-Stage TMLE to assess the effect on the relative scale and keep the matched pairs. In Stage 1, we estimated the community-specific, cumulative HIV incidence $Y^c$ with TMLE adjusting for possibly differential capture of final HIV status. These individual-level TMLEs used Super Learner to combine predictions from penalized regression, generalized additive models, main terms regression, and the empirical mean. In Stage 2, the intervention effect was estimated with a community-level TMLE using Adaptive Prespecification to select the optimal adjustment set from baseline HIV prevalence, baseline male circumcision coverage, or nothing (unadjusted).

A similar approach was taken for all secondary endpoints, including the incidence of HIV-associated TB or death due to illness, hypertension control among adults (30+ years) with baseline hypertension, and population-level HIV viral suppression (HIV RNA$<$500 copies/mL). When assessing the impact on TB or death due to illness, we used the Kaplan-Meier method in Stage 1 to estimate the 3-year risk in each community, separately; we censored at death due to other causes, outmigration, and study close. When assessing the impacts on hypertension control and HIV viral suppression, we implemented individual-level TMLEs in Stage 1 to adjust for baseline and time-varying causes of missingness. For all secondary endpoints, we used a community-level TMLE with Adaptive Prespecification to assess the intervention effect in Stage 2.

As shown in Table 3, the point estimates of the intervention effects are similar, but the precision gains from the Stage 2 approach are notable. Here, “efficiency” is the variance of the unadjusted effect estimator breaking the matches divided by the variance of an alternative approach. For the primary endpoint (HIV incidence), we see precision gains when keeping versus breaking the matches; specifically, the unadjusted effect estimator is 3.1-times more efficient in the pair-matched analysis. As expected, TMLE with Adaptive Prespecification keeping the matches is the most efficient approach and 4.6-times more efficient than the standard approach.

**Table 3.** For selected endpoints in the SEARCH Study, point estimates, 95% confidence intervals, and efficiency comparisons when estimating the intervention effect in Stage 2 with the unadjusted estimator and with TMLE using Adaptive Prespecification. All approaches adjusted for individual-level missingness in Stage 1.

		Breaking the matches		Keeping the matches	
	Stage 2	Effect (95% CI)	Efficiency	Effect (95% CI)	Efficiency
HIV incidence	Unadjusted	0.98 (0.66, 1.45)	1	0.98 (0.78, 1.24)	3.1
	TMLE	0.96 (0.73, 1.26)	2.1	0.96 (0.8, 1.17)	4.6
TB incidence	Unadjusted	0.79 (0.64, 0.98)	1	0.79 (0.69, 0.92)	2.2
	TMLE	0.8 (0.67, 0.95)	1.4	0.8 (0.69, 0.91)	2.6
Hypertension control	Unadjusted	1.19 (1.1, 1.3)	1	1.19 (1.11, 1.28)	1.7
	TMLE	1.18 (1.1, 1.26)	1.6	1.19 (1.11, 1.27)	1.8
Viral suppression	Unadjusted	1.15 (1.11, 1.2)	1	1.15 (1.11, 1.2)	1
	TMLE	1.16 (1.13, 1.2)	1.1	1.15 (1.11, 1.2)	1

Efficiency: Variance estimate for the unadjusted effect estimator breaking the matches used for randomization, divided by the variance estimate of another approach (e.g., TMLE with Adaptive Prespecification, keeping the matches used for randomization).

Similar results are seen for the incidence of HIV-associated TB and hypertension control (Table 3). TMLE using Adaptive Prespecification and keeping the matches is $\approx$2-times more efficient than the unadjusted effect estimator ignoring the matches. In contrast, minimal gains in efficiency are seen when evaluating the effect on HIV viral suppression. This is because the adaptive approach used in TMLE defaults to the unadjusted effect estimator when adjustment does not improve precision. In this scenario, controlling for the baseline prevalence of viral suppression or the proportion of youth (15–24 years) with HIV did not improve precision over the unadjusted effect estimator. However, we note that assuming MCAR and relying on the unadjusted estimator in Stage 1 resulted in vast over-estimation of this endpoint in the intervention arm (85.2% vs. 79.0%) and control arm (75.8% vs. 67.8%) (Table S5 of the Supplementary material available at *Biostatistics* online).

## 6. Discussion

Cluster randomized trials (CRTs) are essential for assessing the effectiveness of interventions delivered to groups of individuals (e.g., clinics or communities). There have been notable advances in the design and conduct of CRTs (e.g., Turner *and others,* 2017a,b; Murray *and others,* 2018, 2020). However, substantial challenges remain and threaten the quality of evidence generated by CRTs. Regardless of best intentions, most CRTs are prone to differential measurement of individual-level outcomes and to covariate imbalance. In this paper, we proposed and evaluated a novel approach, Two-Stage TMLE, to address the dual challenges of bias due to missing individual-level outcomes and imprecision due to few randomized units (i.e., clusters). In Stage 1, an individual-level TMLE is implemented within each cluster separately to estimate a cluster-specific endpoint $Y^c$, which appropriately controls for missingness on participant outcomes. Fully stratifying on the cluster simplifies identification and allows the missingness mechanism to vary by cluster. In Stage 2, the treatment effect is estimated with a separate, cluster-level TMLE to compare the cluster-level endpoints $\hat{Y}^c$, estimated from Stage 1. Adaptive Prespecification is used in Stage 2 to flexibly select from a pre-specified set the adjustment variables and, thus, the TMLE that maximize precision (Balzer *and others,* 2016). Statistical inference is based on the estimated influence curve and the Student’s $t$-distribution. Finite sample simulations demonstrated the potential for Two-Stage to overcome the shortcomings of existing CRT methods, especially when there are post-baseline causes of missingness. Application to real data from the SEARCH Study demonstrated the precision gains attained through adaptive adjustment in Stage 2.

To the best of our knowledge, Two-Stage TMLE is the first CRT estimator that simultaneously addresses bias due to individual-level missingness and improves efficiency through adaptive adjustment for covariate imbalance, in a fully prespecified manner. The approach is applicable to a wide range of measurement schemes (e.g., single cross-sectional sample, repeated cross-sectional sampling, and longitudinal follow-up) and endpoint types (e.g., binary, continuous, time-to-event outcomes). The approach is also applicable to a wide range of causal parameters (e.g., population, conditional, and sample effects) and scales of inference (e.g., absolute or relative measures). Additionally, Two-Stage TMLE should naturally generalize to hierarchical data settings with a nonrandomized, cluster-level exposure. In such an observational setting, the cluster-level TMLE implemented in Stage 2 would focus on confounding control, as opposed to efficiency improvement. However, the asymptotic properties and finite sample performance of such an estimator remain an area of future work.

Altogether, Two-Stage TMLE alleviates, but does not fully resolve, the challenges that arise from missing data in CRTs. Stage 1 uses TMLE with machine learning to flexibly adjust for baseline and time-dependent causes of missingness and, as a plug-in estimator, provides more stability under strong confounding or rare outcomes. However, adjustment for missingness in Stage 1 occurs within each cluster separately, limiting the breadth and flexibility of adjustment when the cluster-specific outcome is rare or the cluster-specific sample size is small (e.g., in subgroup analyses). This highlights a challenge commonly occurring in finite samples: we must balance the strength of our assumptions for identifiability (e.g., MCAR vs. MAR) and for estimation (e.g., parametric regressions vs. Super Learner) with limited data support. In CRTs with very small cluster sizes and highly differential measurement, a single stage TMLE is likely to be more appropriate. However, such an TMLE does yet not exist and is an area of future work.

## Supplementary Material

kxab043_Supplementary_Data
