# Supplementary material for: Two-Stage TMLE to reduce bias and improve efficiency in cluster randomized trials
Source: Biostatistics. 2021 Dec 23;24(2):502–17. doi: 10.1093/biostatistics/kxab043 (PMC10102904; doi:10.1093/biostatistics/kxab043)
Supplement: kxab043_Supplementary_Data [file kxab043_supplementary_data.pdf]

Biostatistics (2021), **0**, 0, *pp.* 1–24
doi:10.1093/biostatistics/Supp

Supplementary Materials: Two-Stage TMLE to Reduce Bias and Improve Efficiency in Cluster Randomized Trials

LAURA B. BALZER*, MARK VAN DER LAAN, JAMES AYIEKO, MOSES KAMYA,
GABRIEL CHAMIE, JOSHUA SCHWAB, DIANE V. HAVLIR, MAYA L. PETERSEN

Summary

In the following Supplementary Materials, we provide: (i) a brief overview of Hierarchical TMLE; (ii) step-by-step implementation of TMLE in Stage 1 to control for differential missingness on individual-level outcomes; (iii) a discussion of causal parameters and their identification in Stage 2; (iv) step-by-step implementation of TMLE in Stage 2 to maximize efficiency when estimating the intervention effect; (v) details on the asymptotic linearity for Two-Stage TMLE; (vi) a second simulation study; (vii) additional results from the main simulation study; (viii) additional results from the real data application, and (ix) computing code.

Key words: Clustered data; Cluster randomized trials; Covariate adjustment; Data-adaptive; Double robust; Group randomized trials; Missing data; Multi-level model; Super Learner; TMLE.

1. Brief Overview of TMLE and of Hierarchical TMLE

The basic steps of targeted minimum loss-based estimation (TMLE) for a point-treatment problem are as follows (van der Laan and Rose, 2011):

1. Estimating the outcome regression: the conditional expectation of the outcome given the

© The Author 2021. Published by Oxford University Press. All rights reserved. For permissions, please e-mail: journals.permissions@oup.com

intervention of interest and the adjustment covariates

2. Estimating the propensity score: the conditional probability of the intervention given the adjustment covariates

3. Targeting the estimator of outcome regression with information in the estimated propensity score

4. Obtaining a point estimate by averaging the targeted predictions of the outcome

5. Obtaining statistical inference (i.e., Wald-Type 95% confidence intervals) with the estimated influence curve

We refer readers Schuler and Rose (2017) and Blakely *and others* (2019) for an introduction. Step-by-step implementation for statistical parameters corresponding to hypothetical interventions on the measurement process and for evaluating the treatment effect are given in Sections 2 and 4, respectively.

Recently, Balzer *and others* (2019) proposed and validated an extension of TMLE for estimation and inference for the effects of cluster-based exposures in observational studies and randomized trials with complete outcome measurement (i.e., no missingness). Briefly, this work explores the theoretical and finite sample performance of 3 different TMLEs:

1. *Cluster-level TMLE:* The cluster-level TMLE is implemented after the data are aggregated to the cluster-level. Initial estimation and targeting of the outcome regression are done at the cluster-level (i.e., with a cluster-level, propensity score estimate).

2. *Hybrid-TMLE:* The Hybrid-TMLE is implemented using both individual-level and cluster-level data. Initial estimation of the outcome regression is done at the individual-level, which naturally harnesses the pairing of individual-level outcomes and baseline covariates. Estimates from this individual-level outcome regression are aggregated to the cluster-level and

targeted with a cluster-level, propensity score.

3. *Individual-level TMLE:* Point estimation for the individual-level TMLE follows a fully individual-level approach; statistical inference, however, respects the cluster as the independent unit. Initial estimation and targeting of the outcome regression are done at the individual-level (i.e., with an individual-level, propensity score estimate).

These approaches are collectively known as "Hierarchical TMLE" (Balzer *and others*, 2019). Recently, Yang (2021) extended Adaptive Pre-specification to select between these TMLEs the one which maximizes the empirical efficiency. Additionally, Benitez *and others* (2021) provide details on how weights can be applied to these TMLEs to estimate a variety of causal effects (e.g., effects at the individual-level and at the cluster-level; overview in Section 3).

Hierarchical TMLE has not yet been generalized to handle missingness on individual-level outcomes in CRTs. If there is no missingness and the individual-level outcome regression is fit within each cluster separately, then the Hybrid-TMLE can be considered to be a special case of Two-Stage TMLE, proposed here. Theoretically and in simulations mimicking the SEARCH Study but with complete outcome measurement, the Hybrid-TMLE dramatically increased efficiency and statistical power over the unadjusted effect estimator, while maintaining Type-I error control (Balzer *and others*, 2019). Therefore, in our Two-Stage approach, adjusting for individual-level covariates in Stage 1 is expected to increase the efficiency for effect estimation in Stage 2. When outcomes are completely measured, we can use Adaptive Pre-specification to select among the following TMLEs the one which maximizes empirical efficiency: (1) the cluster-level TMLE, (2) the Hybrid-TMLE where the individual-level outcome regressions are fit within each cluster separately, (3) the Hybrid-TMLE where the individual-level outcome regression is fit pooling over clusters, and (4) the fully individual-level TMLE. (We again note that approach # 2 would be equivalent to Two-Stage TMLE when there is no missingness.)

Since participant outcomes are missing in over 90% of CRTs (Fiero *and others*, 2016), we

focus the remainder of the Supplementary Materials on Two-Stage TMLE, which simultaneously controls for differential outcome measurement and adjusts for covariate imbalance to reduce bias and improve efficiency in CRTs.

2. Step-by-Step Implementation of TMLE in Stage 1

For demonstration, we focus on implementation of TMLE for the cluster-specific endpoint

$$Y^c \equiv \mathbb{E}\big[\mathbb{E}(Y \big| \Delta = 1, W, M)\big] \tag{2.1}$$

where Y is the individual-level outcome, Δ is an indicator of measurement, W are baseline individual-level covariates, and M are post-intervention individual-level covariates (i.e., mediators). We note that in settings with complex dependence, the adjustment set (W, M) can be expanded to include the baseline and post-intervention covariates each participant's "friends".

To estimate Eq. 2.1 with TMLE, we take the following steps **within each cluster** $i = \{1, \ldots, N\}$**, separately**. Throughout, $j = \{1, \ldots, S_i\}$ indexes the participants of cluster i. For ease of notation, we drop the superscript c when denoting the cluster-size S in the Supplementary Materials.

1. Among those with measured outcomes (i.e., $\Delta = 1$), use Super Learner to flexibly model the relationship between the outcome Y and adjustment variables (W, M).

2. Use the output from #1 to predict the outcome for all participants, regardless of their measurement status: $\hat{\mathbb{E}}(Y \mid \Delta = 1, W_j, M_j)$ for $j = \{1, \ldots, S_i\}$.

3. Target these machine learning-based predictions with information in the estimated measurement mechanism $\hat{\mathbb{P}}(\Delta = 1 \mid W, M)$, also fit with Super Learner.

   (a) Calculate the "clever covariate" $\hat{H}_j = \frac{\mathbb{I}(\Delta_j=1)}{\hat{\mathbb{P}}(\Delta=1|W_j,M_j)}$ for $j = \{1, \ldots, S_i\}$

   (b) Run logistic regression of outcome Y on only the intercept, using the logit of the initial

estimator $\hat{\mathbb{E}}(Y \mid \Delta = 1, W, M)$ as offset (i.e., fixing its coefficient to 1) and the clever covariate $\hat{H}$ as weight.

(c) Denote the resulting intercept as $\hat{\epsilon}$.

4. Obtain targeted predictions of the outcome for all participants, regardless of their measurement status: $\hat{\mathbb{E}}^*(Y \mid \Delta = 1, W_j, M_j)$ for $j = \{1, \ldots, S_i\}$.

   (a) Add the estimated intercept to the logit of the initial estimates and transform back to the original scale (i.e., take the inverse-logit): $\hat{\mathbb{E}}^*(Y \mid \Delta = 1, W_j, M_j) = logit^{-1}\big[\hat{\epsilon} + logit\{\hat{\mathbb{E}}(Y \mid \Delta = 1, W_j, M_j)\}\big]$

5. Average the targeted predictions to obtain an estimate of the cluster-specific endpoint adjusted for missingness on individual-level outcomes:

$$\hat{Y}^c = \frac{1}{S}\sum_{j=1}^{S} \hat{\mathbb{E}}^*(Y \big| \Delta = 1, W_j, M_j)$$

Because we are implementing TMLE in each cluster separately, we do not include the cluster-level covariates E^c or treatment A^c in the above estimation procedure. Updating on the logit-scale is recommended for binary and continuous individual-level outcomes; for details see Gruber and van der Laan (2010).

This Stage 1 approach of identifying and then using TMLE to estimate a cluster-specific endpoint Y^c, which adjusts for differential outcome ascertainment, also applies to more complicated settings, including time-to-event outcomes with differential censoring and when we have missingness on both the characteristic defining the population of interest and on the outcome of interest (Petersen *and others*, 2014; Benkeser *and others*, 2019; Balzer *and others*, 2020). We refer the reader to Section 3.1.1-3.1.2 of the main text for an overview.

3. Stage 2 Causal Parameters & their Identification

Recall our objective is to estimate the effect of the cluster-level intervention with optimal precision, after adjusting for differential missingness on individual-level outcomes. Let $Y(a^c, 1)$ be the individual-level counterfactual outcome, generated by hypothetical interventions to set the cluster-level treatment $A^c = a^c$ and to ensure complete measurement of the individual-level outcomes (i.e., "setting" $\Delta = 1$). As detailed in Benitez *and others* (2021), we can use these individual-level counterfactuals to define a variety of cluster-level and individual-level effects in CRTs. For example, we can define the cluster-level counterfactual outcome as the expectation of the individual-level counterfactual outcomes:

$$Y^c(a^c) \equiv \mathbb{E}[Y(a^c, \delta = 1)] \tag{3.2}$$

The Stage 2 causal parameter is then a summary measure of the distribution of the cluster-level counterfactuals $Y^c(a^c)$. A common target is the population average treatment effect (PATE):

$$\mathbb{E}[Y^c(1)] - \mathbb{E}[Y^c(0)] \tag{3.3}$$

Alternatively, we could be interested in the sample average treatment effect (SATE), which is the effect for the N study clusters (Neyman, 1923; Rubin, 1990), or in summary measures on the relative scale. In the SEARCH Study, for example, the primary analysis was for the sample risk ratio for the $N = 32$ trial communities:

$$\frac{\frac{1}{N}\sum_{i=1}^{N} Y_i^c(1)}{\frac{1}{N}\sum_{i=1}^{N} Y_i^c(0)} \tag{3.4}$$

where $Y_i^c(a^c)$ was the counterfactual cumulative incidence of HIV in community i.

We can consider a wider range of causal parameters by combining each summary measure with weights. Specifically, let S_i be the size of cluster i, and consider a weighted-version of the treatment-specific sample mean: $1/N \sum_i \alpha_i Y_i^c(a^c)$. Then setting $\alpha_i = \frac{S_i \times N}{\sum_i S_i}$ gives equal weight to participants, while setting $\alpha_i = 1$ gives equal weight to clusters (Benitez *and others*, 2021).

When there is an interaction between cluster size and the treatment, cluster size is said to be "informative" (Seaman *and others*, 2014), and the resulting causal parameters will generally not be equivalent. In all settings, the target effect should be pre-specified and be driven by research question. We refer the reader to Benitez *and others* (2021) for a detailed discussion on target causal parameters in CRTs.

Since we have already controlled for missing outcomes in Stage 1, identification of the Stage 2 causal parameter is trivial. Specifically, the randomization assumption ($Y^c(a^c) \perp\!\!\!\perp A^c$) and the positivity assumption ($0 < \mathbb{P}(A^c = 1) < 1$) hold by design in CRTs. Therefore, we can identify PATE as

$$\mathbb{E}[Y^c(1)] - \mathbb{E}[Y^c(0)] = \mathbb{E}(Y^c|A^c = 1) - \mathbb{E}(Y^c|A^c = 0) \tag{3.5}$$

where Y^c denotes the Stage 1 estimand, which appropriately adjusts for missingness on individual-level outcomes (e.g., Eq. 2.1 of the Supplementary Materials). This framework for specifying and identifying causal effects in Stage 2 also applies for more complicated Stage 1 endpoints, corresponding to different Y^cs. (See Sections 3.1.1-3.1.2 of the main text.)

As repeatedly demonstrated (e.g., Gail *and others* (1996); Moore and van der Laan (2009); Rosenblum and van der Laan (2010); Colantuoni and Rosenblum (2015); Turner *and others* (2017); Murray *and others* (2020); Benkeser *and others* (2020)), adjustment for baseline covariates can improve precision in randomized trials. Therefore, our statistical estimand corresponding to the treatment-specific, population mean $\mathbb{E}[Y^c(a^c)]$ is given by

$$\psi(a^c) \equiv \mathbb{E}\big[\mathbb{E}(Y^c|A^c = a^c\big|E^c, W^c)\big] \tag{3.6}$$

Likewise, our statistical estimand for the PATE is $\psi(1) - \psi(0)$. Of course, we can also take the ratio of $\psi(1)$ and $\psi(0)$ to obtain a relative effect. Identification of causal parameters for the corresponding sample and conditional effects is discussed in Balzer *and others* (2016*a*).

4. Step-by-Step Implementation of the TMLE in Stage 2

Given estimates of the cluster-specific endpoints $\hat{Y}_i^c$ for $i = \{1, \ldots, N\}$ from Stage 1, we then implement a cluster-level TMLE to more efficiently estimate the intervention effect in Stage 2. For demonstration, we focus on TMLE for relative effect: $\psi(1)/\psi(0)$.

1. Obtain an initial estimate of the conditional expectation of the cluster-level outcome, given the cluster-level treatment and covariates: $\hat{\mathbb{E}}(\hat{Y}^c|A^c, E^c, W^c)$. We could, for example, fit a "working" regression of the estimated outcome $\hat{Y}^c$ on an intercept with main terms for the cluster-level treatment A^c and selected cluster-level covariates (E^c, W^c) (Moore and van der Laan, 2009; Rosenblum and van der Laan, 2010).

2. Use the output from #1 to predict the outcome for all clusters under both the intervention and control conditions: $\hat{\mathbb{E}}(\hat{Y}^c|A^c = 1, E_i^c, W_i^c)$ and $\hat{\mathbb{E}}(\hat{Y}^c|A^c = 0, E_i^c, W_i^c)$ for $i = \{1, \ldots, N\}$.

3. Target the initial predictions using information in the estimated propensity score $\hat{\mathbb{P}}(A^c = 1|E_i^c, W_i^c)$ for $i = \{1, \ldots, N\}$.

   (a) To estimate the cluster-level propensity score, we could again fit a "working" logistic regression of the cluster-level treatment indicator A^c on an intercept and selected cluster-level covariates (E^c, W^c).

   (b) Calculate the two-dimensional "clever" covariate: $\hat{H}1_i^c = \frac{\mathbb{I}(A_i^c=1)}{\hat{\mathbb{P}}(A^c=1|E_i^c,W_i^c)}$ and $\hat{H}0_i^c = \frac{\mathbb{I}(A_i^c=0)}{\hat{\mathbb{P}}(A^c=0|E_i^c,W_i^c)}$ for $i = \{1, \ldots, N\}$.

   (c) Run logistic regression of cluster-level outcome $\hat{Y}^c$ on the clever covariates $\hat{H}1^c$ and $\hat{H}0^c$, suppressing the intercept, and using the logit of the initial estimator $\hat{\mathbb{E}}(\hat{Y}^c|A^c, E^c, W^c)$ as offset (i.e., fixing its coefficient to 1).

   (d) Denote the resulting coefficient estimates corresponding to $\hat{H}1^c$ and $\hat{H}0^c$ as $\hat{\epsilon}1^c$ and $\hat{\epsilon}0^c$, respectively.

4. Obtain targeted predictions of the outcome for all clusters under both the intervention and control conditions:

$$\hat{\mathbb{E}}^*(\hat{Y}^c|A^c=1,E^c,W^c) = logit^{-1}\big[logit\{\hat{\mathbb{E}}(\hat{Y}^c|A^c=1,E^c,W^c)\} + \hat{\epsilon}1^c/\hat{\mathbb{P}}(A^c=1|E^c,W^c)\big]$$
$$\hat{\mathbb{E}}^*(\hat{Y}^c|A^c=0,E^c,W^c) = logit^{-1}\big[logit\{\hat{\mathbb{E}}(\hat{Y}^c|A^c=0,E^c,W^c)\} + \hat{\epsilon}0^c/\hat{\mathbb{P}}(A^c=0|E^c,W^c)\big]$$

5. Obtain a point estimate by dividing the average of the targeted predictions under the intervention condition by the average of the targeted predictions under the control condition:
$$TMLE = \frac{\hat{\psi}^*(1)}{\hat{\psi}^*(0)} = \frac{\frac{1}{N}\sum_{i=1}^N \hat{\mathbb{E}}^*(\hat{Y}^c \mid A^c=1,E_i^c,W_i^c)}{\frac{1}{N}\sum_{i=1}^N \hat{\mathbb{E}}^*(\hat{Y}^c \mid A^c=0,E_i^c,W_i^c)}$$

If the known propensity score is not estimated (e.g., $\mathbb{P}(A^c=1)=0.5$ in two-armed CRTs with balanced allocation), then the targeting step can be skipped. As detailed in Moore and van der Laan (2009), using a two-dimensional clever covariate during updating (step 3) allows for simultaneous targeting of the treatment-specific means and effects on the additive, relative, and odds ratio scales. As detailed in Balzer *and others* (2016*a*), implementation to obtain a point estimate is identical for the population, conditional, and sample effects.

To flexibly select among various estimators of the outcome regression and propensity score, we recommend using *Adaptive Pre-specification*, as described in the main text and detailed in Balzer *and others* (2016*b*).

5. Asymptotic Linearity of Two-Stage TMLE

Briefly, an estimator is asymptotically linear if the difference between the estimator and the estimand behaves (in first order) as an empirical average of a mean-zero and finite variance function, known as the influence curve, of the unit data (Bickel *and others*, 1993; van der Vaart and Wellner, 1996; van der Laan and Rose, 2011). An asymptotically linear estimator will be consistent and normally distributed in its limit. Therefore, the Central Limit Theorem can be applied to construct 95% confidence intervals and test the null hypothesis.

Recall that in Stage 1, we first define the cluster-specific outcome Y^c. If all individual-level outcomes are completely measured, then Y^c could be defined as the expected individual-level outcome within each cluster: $\mathbb{E}[Y]$. If the individual-level outcomes are missing-completely-at-random (MCAR), then Y^c could be defined as the expected individual-level outcome among those measured: $\mathbb{E}[Y|\Delta = 1]$. Likewise, if measurement Δ depends on individual-level, baseline covariates W, then Y^c could be defined as the expected individual-level outcome given measurement and those covariates, standardized with respect to the covariate distribution: $\mathbb{E}\big[\mathbb{E}(Y|\Delta = 1, W)\big]$. Extensions to scenarios with post-baseline causes of missingness and/or right-censoring follow analogously.

Next, we estimate the cluster-specific outcome Y_i^c within each cluster $i = \{1, \ldots, N\}$, separately. When outcomes are completely measured ($Y^c = \mathbb{E}[Y]$) or are missing-completely-at-random ($Y^c = \mathbb{E}[Y|\Delta = 1]$), a simple and intuitive estimator is the empirical mean outcome among those measured. When outcomes are missing-at-random within values of the adjustment variables (e.g., $Y^c = \mathbb{E}[\mathbb{E}(Y|\Delta = 1, W)]$), we recommend using TMLE with Super Learner for estimation of the cluster-specific outcome. The empirical mean outcome (among those measured) can be considered a special case of TMLE where the adjustment set is empty: $W = \{\}$.

To emphasize how the Stage 1 estimator depends on the individual-level data within each cluster, let P_i denote the true distribution of the individual-level data in cluster i. Likewise, let P_{i,S_i} denote the targeted estimator of that distribution based on S_i individuals in cluster i. Then we can write the Stage 1 cluster-specific estimand as $Y_i^c \equiv \Phi^c(P_i)$ and the Stage 1 cluster-specific plug-in estimator as $\hat{Y}_i^c \equiv \Phi^c(P_{i,S_i})$.

The Stage 2 cluster-level effect estimator is, therefore, a function of $\Phi^c(P_{i,S_i})$, $i = \{1, \ldots, N\}$. Consider, for example, the treatment-specific mean $\mathbb{E}[Y^c(a^c)]$ as our Stage 2 target parameter. Then the cluster-level TMLE of the corresponding statistical estimand $\psi(a^c) = \mathbb{E}[\mathbb{E}(Y^c|a^c, E^c)]$

in Stage 2 would be

$$\hat{\psi}(a^c) = \frac{1}{N}\sum_{i=1}^{N} \hat{\mathbb{E}}^*(\hat{Y}^c|A^c = a^c, E_i^c) = \frac{1}{N}\sum_{i=1}^{N} \hat{\mathbb{E}}^*\left(\Phi^c(P_{i,S_i})|A^c = a^c, E_i^c\right)$$

(For ease of notation, we use E^c to represent both the cluster-level covariates and aggregates of individual-level covariates (i.e. W^c) in this sub-section.) An unadjusted effect estimator in Stage 2 can again be considered a special case of the cluster-level TMLE where the adjustment set is empty: $E^c = \{\}$.

Under the following conditions, Two-Stage TMLE will be asymptotically linear, meaning that

$$\hat{\psi}(a^c) - \psi(a^c) = \frac{1}{N}\sum_{i=1}^{N} D_i + R_N$$

where D_i represents the cluster-level influence curve and $R_N = o_P(N^{-1/2})$ is remainder term, going to zero in probability:

1. Stage 2 estimators of the cluster-level outcome regression and the cluster-level propensity score meet the usual regularity conditions, which are quite weak in a randomized trial (e.g., Moore and van der Laan (2009); Rosenblum and van der Laan (2010)).

2. Deviations between the estimated cluster-level outcomes and the true cluster-level outcomes, $\frac{1}{\sqrt{N}}\sum_{i=1}^{N} \Phi^c(P_{i,S_i}) - \Phi^c(P_i)$, provide a negligible contribution to the remainder term R_N.

The conditions on Stage 2 estimation are satisfied when estimating the known, cluster-level propensity score with a "working" logistic regression and when estimating the cluster-level outcome regression with another "working" parametric regression (e.g., Moore and van der Laan (2009); Rosenblum and van der Laan (2010)). However, to the best of our knowledge, all previously existing Two-Stage estimators (e.g., a t-test on the cluster-level means) have simply ignored the contribution from estimating the cluster-level outcome to R_N. Suppose, for example, our Stage 1 estimator is the average outcome within each cluster: $\Phi^c(P_{i,S_i}) = \hat{\mathbb{E}}_{P_{i,S_i}}(Y|\Delta = 1)$.

(Such an estimator would only be appropriate when the individual-level outcomes are completely measured or are missing-completely-at-random.) Since the individual-level outcomes are not i.i.d. within each cluster, we need the following to hold for this estimator's contribution to R_N to be essentially zero: (1) the within cluster dependence is weak enough that the Central Limit Theorem applies in S_i, and (2) the smallest cluster is much larger than the total number of clusters (i.e., $N/min_i(S_i) \to 0$).

When the Stage 1 estimator $\Phi^c(P_{i,S_i})$ is a TMLE of the Stage 1 estimand $\Phi^c(P_i)$, the relevant component of the remainder term R_N can be written as

$$\frac{1}{\sqrt{N}} \sum_{i=1}^{N} \Big[(P_{i,S_i} - P_i) D^*_{i,P_{i,S_i}} + R_i(P_{i,S_i}, P_i) \Big] \tag{5.7}$$

where $D^*_{i,P_{i,S_i}}$ and $R_i(P_{i,S_i}, P_i)$ are the cluster i-specific efficient influence curve and remainder terms, respectively. As before, we need that the within cluster dependence is weak enough such that $(P_{i,S_i} - P_i) D^*_{i,P_{i,S_i}} = O_P(S_i^{-1/2})$ and that the ratio of total number of clusters to the cluster-size goes to zero (i.e., $N/min_i(S_i) \to 0$). We note that when the cluster-size S_i is substantially larger than N, we can weaken this independence assumption to allow for a slower rate of convergence. Additionally, we need that estimators of the individual-level outcome regression and the individual-level missingness mechanism converge to their targets at fast enough rates such that $R_i(P_{i,S_i}, P_i) = o_P(S_i^{-1/2})$ (van der Laan and Rose, 2011). Implementing Super Learner with highly adaptive LASSO (HAL) (Benkeser and van der Laan, 2016) or internal sample-splitting can help ensure these conditions hold in practice (Zheng and van der Laan, 2011; Díaz, 2019).

5.1 *Inference for Pair-matched Trials*

This approach to statistical inference also applies in CRTs where the treatment is randomized within matched pairs of clusters. Briefly, let O^c_{k1} and O^c_{k2} denote the observed data for the first and second cluster within matched pair k, respectively. To obtain statistical inference for the effect in a pair-matched setting, we replace $\hat{D}(O^c)$ with the following paired version: $\hat{D}_{paired}(O^c_{k1}, O^c_{k2}) =$

$\frac{1}{2}\left[\hat{D}(O_{k1}^c) + \hat{D}(O_{k2}^c)\right]$ (Balzer *and others*, 2016*a*). Our variance estimator is then given by the sample variance of the paired influence curve divided by the number of pairs ($N/2$), and we use the Student's t-distribution with $N/2 - 1$ degrees of freedom (Hayes and Moulton, 2009). This could naturally be extended to matched triplets in a three-armed trial.

6. Additional Simulation Study with Baseline (only) Causes of Missingness

Here, we consider a simplified scenario where only baseline (but not post-baseline) covariates impact the measurement of individual-level outcomes. As before, we focus on a setting with $N = 30$ clusters and where within each cluster, the number of individual participants is sampled with equal probability from {100, 150, 200}.

For each cluster $i = \{1, \ldots, N\}$, we independently generate the cluster-specific data as follows. First, one latent variable $U1^c$ is drawn uniformly from (1.75, 2.25) and two additional variables $(U2^c, U3^c)$ are drawn independently from a standard normal distribution. Then, two individual-level covariates $(W1, W2)$ are generated by drawing from a normal distribution with means depending on the cluster-level latent factors: $W1 \sim Norm(U1^c, 1)$ and $W2 \sim Norm(U2^c, 1)$. We set the observed cluster-level covariates $(E1^c, E2^c)$ as the empirical mean of their individual-level counterparts. The intervention A^c is randomly allocated within pairs of clusters matched on $U3^c$; therefore, $N/2$ clusters receive the intervention and $N/2$ the control.

The underlying, individual-level outcome Y is generated as an indicator that U_Y, drawn from a Uniform(0,1), is less than $logit^{-1}\{-4 + 0.15A^c + 0.15A^cW1 + 0.4W1 + 0.2W2 + 0.5E1^cW1 + 0.3(E1^c + E2^c + U3^c)\}$. Finally, we incorporate individual-level missingness by generating Δ as an indicator that U_Δ, drawn from a Uniform(0,1), is less than $logit^{-1}(4 - 0.25A^c - 0.75A^cW1 - 0.75W1 - 0.1W2 - 0.5E1^c - 0.1E2^c)$. Thus, participants in the intervention arm ($A^c = 1$), and especially those with higher values of $W1$, are more likely to have the outcome and also be missing. The observed outcomes Y are set to be missing for individuals with $\Delta = 0$.

We also generate the counterfactual, individual-level outcomes $Y(1,1)$ and $Y(0,1)$ by setting the cluster-level treatment to $A^c = 1$ and $A^c = 0$, respectively, and preventing missingness by setting $\Delta = 1$. As before, the cluster-level counterfactual outcome is the empirical mean of the individual-level counterfactual outcomes within each cluster $Y^c(a^c) \equiv 1/S_i \sum_{i=1}^{S_i} Y_i(a^c, 1)$. The true values of the treatment-specific, population means $\mathbb{E}[Y^c(a^c)]$ for $a^c = \{1, 0\}$, their difference, and their ratio are calculated for a population of 5000 clusters. We compare the same estimators as the main simulation study.

6.1 *Results from the Second Simulation Study*

In this simulation study, the average coefficient of variation was 0.27 in the intervention arm and 0.33 in the control, reflecting higher than expected levels of dependence within clusters (Hayes and Moulton, 2009). The true values of the treatment-specific means were $\mathbb{E}[Y^c(1)]$=47.4% and $\mathbb{E}[Y^c(0)]$=39.6%. The corresponding risk difference and risk ratio were 7.7% and 1.20, respectively. For both effects, Table 1 illustrates estimator performance in this simplified setting.

Focusing first on estimating the risk difference (true value=7.7%), we see that t-test, which fails to adjust for any covariates, is highly biased, as expected given the differential measurement process. On average, it grossly underestimates the intervention effect by 12.4% and attains a confidence interval coverage of <20%, much lower than the nominal rate of 95%. By adjusting for covariates that influence measurement and underlying outcomes, CARE is less biased, but still underestimates the intervention effect by 2.8% when breaking the matches and by 5.1% when preserving the matches. The corresponding confidence interval coverages for CARE are less than the nominal rate: 90.2% and 41.2%, respectively. In contrast, the bias of Two-Stage TMLE for the risk difference is negligible, and the confidence interval coverage is good (>95%). As predicted by theory (Balzer *and others*, 2015), higher power is achieved when preserving, as compared to breaking, the matches: 57.2% versus 46.8%, respectively.

Now focusing on estimating the risk ratio (true value=1.2), we see that both mixed models and GEE overestimate the intervention effect. This bias is substantial enough to prevent accurate inference. The confidence interval coverage is 39.6%-40.6% for mixed models and 22.4%-36.4% for GEE. Lower coverage for GEE is likely due to underestimation of the standard errors ($\hat{\sigma} < \sigma$). While both mixed models and GEE are adjusting for the appropriate variables, both are relying on a misspecified regressions.

Theoretically, DR-GEE should reduce bias from GEE by incorporating estimates of the missingness mechanism. Indeed, DR-GEE exhibits lower bias, but still does not obtain valid inference (confidence interval coverage of 54%). This again highlights the need for flexible (i.e., data-adaptive) estimators of the individual-level outcome regression and measurement mechanism. In contrast, Two-Stage TMLE for the risk ratio is essentially unbiased and confidence interval coverage is good (>95%). Again, more power is achieved when preserving (59%) versus breaking the matched (46.8%).

Table 1. *Over 500 simulated trials each with $N = 30$ clusters, the performance of CRT estimators* ***when missingness is only impacted by baseline variables*** *(i.e., the supplemental simulation study). Results are shown when the target of inference is the risk difference (top 3 rows), when the target is the risk ratio (bottom 4 rows), when breaking the matches during analysis (left), and when preserving the matches during analysis (right).*

	BREAKING THE MATCHES						KEEPING THE MATCHES					
	$\hat{pt}$	bias	σ	$\hat{\sigma}$	CI	power	$\hat{pt}$	bias	σ	$\hat{\sigma}$	CI	power
	FOR THE RISK DIFFERENCE (true value RD=7.7%)											
t-test	-4.6	-12.4	0.040	0.043	19.4	15.8	-4.6	-12.4	0.040	0.039	18.2	22.6
CARE	4.9	-2.8	0.019	0.028	90.2	32.0	2.6	-5.1	0.013	0.023	41.2	1.8
TMLE	7.2	-0.6	0.023	0.036	99.4	46.8	7.2	-0.5	0.023	0.031	98.8	57.2
	FOR THE RISK RATIO (true value RR=1.20)											
Mixed	1.7	0.5	0.148	0.144	40.6	90.4	1.7	0.5	0.142	0.144	39.6	92.0
GEE	1.6	0.5	0.148	0.135	36.4	90.8	1.7	0.5	0.171	0.097	22.4	95.6
DR-GEE	1.4	0.2	0.099	0.085	54.0	92.8						
TMLE	1.2	-0.0	0.053	0.084	99.6	46.8	1.2	-0.0	0.053	0.072	99.0	59.0

$\hat{pt}$: average point estimate (in % for the RD)
bias: average deviation in the point estimates vs. true effect (in % for the RD)
σ: standard deviation of the point estimates (on log-scale for RR)
$\hat{\sigma}$: average standard error estimate (on log-scale for RR)
CI: proportion of 95% confidence intervals containing the true effect (in %)
power: proportion of trials correctly rejecting the false null hypothesis (in %)

7. Main Simulation Study - Additional Results

In the following Tables, we provide additional results from the main simulation study, where both baseline and post-baseline variables (W, M) impact individual-level measurement and outcomes.

In Table 2, we provide the results for the main simulation study when CARE, mixed models, GEE, and DR-GEE include the mediator M in their adjustment set. As expected, forcing adjustment for a variable impacted by the intervention (but also confounds the measurement-outcome relationship) does not serve to eliminate bias due to missing individual-level outcomes.

Table 2. *Over 500 simulated trials each with* $N = 30$ *clusters, the performance of CRT estimators* ***when missingness depends on baseline and post-baseline variables*** *(i.e., the main simulation study) and* ***the mediator*** M ***is included in the adjustment set for CARE, mixed models, GEE, and DR-GEE****. Results are shown when the target of inference is the risk difference (top 3 rows), when the target is the risk ratio (bottom 4 rows), when breaking the matches during analysis (left), and when preserving the matches during analysis (right).*

	BREAKING THE MATCHES						KEEPING THE MATCHES					
	$\hat{pt}$	bias	σ	$\hat{\sigma}$	CI	power	$\hat{pt}$	bias	σ	$\hat{\sigma}$	CI	power
	FOR THE RISK DIFFERENCE (true value RD=-9.1%)											
t-test	-32.0	-22.9	0.048	0.050	0.8	100.0	-32.0	-22.9	0.048	0.047	0.6	100.0
CARE	-17.7	-8.6	0.031	0.028	17.0	100.0	-15.4	-6.4	0.040	0.033	56.0	98.4
TMLE	-9.8	-0.7	0.038	0.046	98.8	52.8	-9.9	-0.8	0.037	0.043	96.6	57.4
	FOR THE RISK RATIO (true value RR=0.88)											
Mixed	0.8	-0.1	0.040	0.064	54.8	99.8	0.8	-0.1	0.040	0.064	54.2	99.8
GEE	0.8	-0.1	0.040	0.043	17.4	100.0	0.8	-0.1	0.047	0.033	3.4	99.8
DR-GEE	0.7	-0.2	0.054	0.064	0.0	100.0						
TMLE	0.9	-0.0	0.051	0.063	98.4	52.6	0.9	-0.0	0.051	0.058	96.8	57.8

$\hat{pt}$: average point estimate (in % for the RD)
bias: average deviation in the point estimates vs. true effect (in % for the RD))
σ: standard deviation of the point estimates (on log-scale for RR)
$\hat{\sigma}$: average standard error estimate (on log-scale for RR)
CI: proportion of 95% confidence intervals containing the true effect (in %)
power: proportion of trials correctly rejecting the false null hypothesis (in %)

To assess performance with fewer clusters, we repeated the main simulation study with $N = 20$ clusters. The results are given in Table 3 and echo the main findings. Even with limited numbers of clusters, Two-Stage TMLE essentially eliminates bias due to differential outcome measurement and achieves nominal confidence interval coverage ($\geqslant$95%). Existing estimators exhibit substantial bias and yield misleading inferences. (Here, the mediator M is not included in the adjustment

sets for CARE, mixed models, GEE, and DR-GEE.)

Table 3. *Over 500 simulated trials, the performance of CRT estimators* ***when missingness depends on baseline and post-baseline variables*** *(i.e., the main simulation study) and* ***there are only*** $N = 20$ ***clusters****. Results are shown when the target of inference is the risk difference (top 3 rows), when the target is the risk ratio (bottom 4 rows), when breaking the matches during analysis (left), and when preserving the matches during analysis (right).*

	BREAKING THE MATCHES						KEEPING THE MATCHES					
	$\hat{pt}$	bias	σ	$\hat{\sigma}$	CI	power	$\hat{pt}$	bias	σ	$\hat{\sigma}$	CI	power
	FOR THE RISK DIFFERENCE (true value RD=-9.1%)											
t-test	-32.4	-23.4	0.059	0.061	7.4	100.0	-32.4	-23.4	0.059	0.058	7.2	100.0
CARE	-21.3	-12.2	0.048	0.044	26.2	99.4	-17.9	-8.8	0.063	0.049	65.2	87.4
TMLE	-9.9	-0.8	0.047	0.054	95.8	39.0	-9.9	-0.8	0.048	0.051	95.2	37.6
	FOR THE RISK RATIO (true value RR=0.88)											
Mixed	0.7	-0.2	0.067	0.086	24.4	98.8	0.7	-0.2	0.067	0.082	22.2	99.0
GEE	0.7	-0.2	0.067	0.069	17.0	98.4	0.7	-0.2	0.076	0.046	5.4	99.2
DR-GEE	0.7	-0.2	0.064	0.064	2.0	99.4						
TMLE	0.9	-0.0	0.064	0.074	96.2	40.6	0.9	-0.0	0.065	0.069	95.2	38.4

$\hat{pt}$: average point estimate (in % for the RD)
bias: average deviation in the point estimates vs. true effect (in % for the RD)
σ: standard deviation of the point estimates (on log-scale for RR)
$\hat{\sigma}$: average standard error estimate (on log-scale for RR)
CI: proportion of 95% confidence intervals containing the true effect (in %)
power: proportion of trials correctly rejecting the false null hypothesis (in %)

To assess Type-I error control for Two-Stage TMLE, we repeated the main simulation study when there was no treatment effect (RD=0; RR=1). The results are given in Table 4 and demonstrate for $N = \{20, 30, 50\}$ clusters, Two-Stage TMLE maintains nominal Type-I error control ($\leqslant$5%)

8. Additional Results from the SEARCH Study

The full statistical analysis plan for the SEARCH Study is available at Balzer *and others* (2018). In Table 5, we provide a comparison of results when using an unadjusted estimator in Stage 1 and Stage 2 versus Two-Stage TMLE when estimating population-level HIV viral suppression (the proportion of all persons with HIV who are suppressing viral replication <500 copiess/mL) in each arm and corresponding the intervention effect (Balzer *and others*, 2020).

Table 4. *Over 500 simulated trials, the performance of Two-Stage TMLE (only)* ***when missingness depends on baseline and post-baseline variables*** *(i.e., the main simulation study) and* ***there is no intervention effect*** *(i.e., under the null). Results are shown for* $N = \{20, 30, 50\}$ *clusters when the target of inference is the risk difference (top), when the target is the risk ratio (bottom), when breaking the matches during analysis (left), and when preserving the matches during analysis (right).*

	BREAKING THE MATCHES						KEEPING THE MATCHES					
	$\hat{pt}$	bias	σ	$\hat{\sigma}$	CI	α	$\hat{pt}$	bias	σ	$\hat{\sigma}$	CI	α
	FOR THE RISK DIFFERENCE (true value RD=0%)											
$N = 20$ clusters	-0.3	-0.3	0.046	0.050	95.6	4.4	-0.3	-0.3	0.046	0.045	95.0	5.0
$N = 30$ clusters	-0.5	-0.5	0.035	0.043	97.6	2.4	-0.5	-0.5	0.035	0.039	95.8	4.2
$N = 50$ clusters	-0.6	-0.6	0.026	0.034	98.4	1.6	-0.6	-0.6	0.026	0.031	97.6	2.4
	FOR THE RISK RATIO (true value RR=1.0)											
$N = 20$ clusters	1.0	-0.0	0.065	0.070	95.8	4.2	1.0	-0.0	0.065	0.064	95.0	5.0
$N = 30$ clusters	1.0	-0.0	0.049	0.060	97.6	2.4	1.0	-0.0	0.050	0.055	95.8	4.2
$N = 50$ clusters	1.0	-0.0	0.037	0.048	98.4	1.6	1.0	-0.0	0.037	0.044	97.4	2.6

$\hat{pt}$: average point estimate (in % for the RD)
bias: average deviation between $\hat{pt}$ & true effect (in % for the RD)
σ: standard deviation of the point estimates (on log-scale for RR)
$\hat{\sigma}$: average standard error estimate (on log-scale for RR)
CI: proportion of 95% confidence intervals containing the true effect (in %)
α: proportion of trials incorrectly rejecting the true null hypothesis (in %)

Table 5. *Summary of arm-specific and effect measures for population-level HIV viral suppression in the SEARCH Study. Point estimates and 95% confidence intervals are provided when assuming MCAR in Stage 1 and using an unadjusted effect estimator in Stage 2 ("Unadjusted") versus when using Two-Stage TMLE to control for missing individual-level outcomes and improve efficiency when estimating the intervention effect ("TMLE"), both when breaking the matches used for randomization and keeping the matches.*

**Estimator**	Intervention (95% CI)	Control (95% CI)	Breaking matches Effect (95% CI)	Keeping matches Effect (95% CI)
Unadjusted	85.2% (83.5%, 86.8%)	75.8% (73.5%, 78.2%)	1.12 (1.08, 1.16)	1.12 (1.09, 1.16)
TMLE	79% (77.1%, 80.8%)	67.8% (66.2%, 69.5%)	1.16 (1.13, 1.2)	1.15 (1.11, 1.2)

9. Computing code

All simulations were conducted in `R` (v4.0.3) using the `nbpMatching`, `lme4`, `geepack`, `CRTgeeDR` `ltmle`, and `SuperLearner` packages (R Core Team, 2020; Beck *and others*, 2016; Bates *and others*, 2015; Hojsgaard *and others*, 2006; Prague *and others*, 2017; Schwab *and others*, 2017; Polley *and others*, 2018). Computing code to reproduce the simulation study is available at `https://github.com/LauraBalzer/TwoStageTMLE`. Computing code used to analyze the SEARCH Study data is available at `https://github.com/LauraBalzer/SEARCH_Analysis_Adults`.

References

Balzer, L.B., Ayieko, J., Kwarisiima, D., Chamie, G., Charlebois, E.D. *and others*. (2020). Far from MCAR: obtaining population-level estimates of HIV viral suppression. *Epidemiology* **31**(5), 620–627.

Balzer, L.B., Havlir, D.V., Schwab, J., van der Laan, M.J., Petersen, M.L. and the SEARCH Collaboration. (2018). Statistical analysis plan for SEARCH phase I: Health outcomes among adults. *Technical Report*, arXiv.

Balzer, L.B., Petersen, M.L. and van der Laan, M.J. (2016*a*). Targeted estimation and inference of the sample average treatment effect in trials with and without pair-matching. *Statistics in Medicine* **35**(21), 3717–3732.

Balzer, L.B., Petersen, M.L., van der Laan, M.J. and the SEARCH Consortium. (2015). Adaptive pair-matching in randomized trials with unbiased and efficient effect estimation. *Statistics in Medicine* **34**(6), 999–1011.

Balzer, L., van der Laan, M., Petersen, M. and the SEARCH Collaboration. (2016*b*). Adaptive pre-specification in randomized trials with and without pair-matching. *Statistics in Medicine* **35**(10), 4528–4545.

Balzer, L.B., Zheng, W., van der Laan, M.J., Petersen, M.L. and the SEARCH Collaboration. (2019). A new approach to hierarchical data analysis: Targeted maximum likelihood estimation for the causal effect of a cluster-level exposure. *Stat Meth Med Res* **28**(6), 1761–1780.

Bates, D., Mächler, M., Bolker, B. and Walker, S. (2015). Fitting linear mixed-effects models using lme4. *Journal of Statistical Software* **67**(1), 1–48.

Beck, C., Lu, B. and Greevy, R. (2016). *nbpMatching: functions for optimal non-bipartite optimal matching*. R package version 1.5.0.

Benitez, A., Petersen, M.L., van der Laan, M., Santos, N., Butrick, E., Walker, D., Ghosh, R., Otieno, P., Waiswa, P. and Balzer, L.B. (2021). Comparative methods for the analysis of cluster randomized trials. *Technical Report*, arXiv.

Benkeser, D., Díaz, I., Luedtke, A., Segal, J., Scharfstein, D. and Rosenblum, M. (2020). Improving precision and power in randomized trials for COVID-19 treatments using covariate adjustment, for binary, ordinal, and time-to-event outcomes. *Biometrics* **Early view**.

Benkeser, D., Gilbert, P.B. and Carone, M. (2019). Estimating and testing vaccine sieve effects using machine learning. *J Am Stat Assoc* **114**(527), 1038–1049.

Benkeser, D. and van der Laan, M. (2016). The highly adaptive lasso estimator. *Proc Int Conf Dat Sci Adv Anal*, 689–696.

Bickel, P.J., Klaassen, C.A.J., Ritov, Y. and Wellner, J.A. (1993). *Efficient and Adaptive Estimation for Semiparametric Models*. Baltimore: Johns Hopkins University Press.

Blakely, T., Lynch, J., Simons, K., Bentley, R. and Rose, S. (2019). Reflection on modern methods: when worlds collide - prediction, machine learning and causal inference. *International Journal of Epidemiology* **dyz132**, 1–7.

Colantuoni, E. and Rosenblum, M. (2015). Leveraging prognostic baseline variables to gain precision in randomized trials. *Stat Med* **34**(2602-2617).

Díaz, I. (2019). Machine learning in the estimation of causal effects: targeted minimum loss-based estimation and double/debiased machine learning. *Biostatistics* **kxz042**.

Fiero, M.H., Huang, S., Oren, E. and Bell, M.L. (2016). Statistical analysis and handling of missing data in cluster randomized trials: a systematic review. *Trials* **17**.

Gail, M.H., Mark, S.D., Carroll, R.J., Green, S.B. and Pee, D. (1996). On design considerations and randomization-based inference for community intervention trials. *Stat Med* **15**, 1069–1092.

Gruber, S. and van der Laan, M.J. (2010). A targeted maximum likelihood estimator of a causal effect on a bounded continuous outcome. *The International Journal of Biostatistics* **6**(1), Article 26.

Hayes, R.J. and Moulton, L.H. (2009). *Cluster Randomised Trials*. Boca Raton: Chapman & Hall/CRC.

Hojsgaard, S., Halekoh, U. and Yan, J. (2006). The R package geepack for generalized estimating equations. *J Stat Softw* **15**(2), 1–11.

Moore, K.L. and van der Laan, M.J. (2009). Covariate adjustment in randomized trials with binary outcomes: Targeted maximum likelihood estimation. *Statistics in Medicine* **28**(1), 39–64.

Murray, D.M., Taljaard, M., Turner, E.L. and George, S.M. (2020). Essential ingredients and innovations in the design and analysis of group-randomized trials. *Annu Rev Public Health* **41**, 1–19.

Neyman, J. (1923). Sur les applications de la theorie des probabilites aux experiences agricoles: Essai des principes (In Polish). English translation by D.M. Dabrowska and T.P. Speed (1990). *Statistical Science* **5**, 465–480.

Petersen, M.L., Schwab, J., Gruber, S., Blaser, N., Schomaker, M. and van der Laan, M.J. (2014). Targeted maximum likelihood estimation for dynamic and static longitudinal marginal structural working models. *Journal of Causal Inference* **2**(2).

Polley, E., LeDell, E., Kennedy, C. and van der Laan, M. (2018). *SuperLearner: Super Learner Prediction*. R package version 2.0-24.

Prague, M., Wang, R., and De Gruttola, V. (2017). CRTgeeDR: an R package for doubly robust generalized estimating equations estimations in cluster randomized trials with missing data. *The R Journal* **9**(2), 105–115.

R Core Team. (2020). *R: A Language and Environment for Statistical Computing*. R Foundation for Statistical Computing, Vienna, Austria.

Rosenblum, M. and van der Laan, M.J. (2010). Simple, efficient estimators of treatment effects in randomized trials using generalized linear models to leverage baseline variables. *The International Journal of Biostatistics* **6**(1), Article 13.

Rubin, D.B. (1990). Comment: Neyman (1923) and causal inference in experiments and observational studies. *Statistical Science* **5**(4), 472–480.

Schuler, M.S. and Rose, S. (2017). Targeted maximum likelihood estimation for causal inference in observational studies. *American Journal of Epidemiology* **185**(1), 65–73.

Schwab, J., Lendle, S., Petersen, M. and van der Laan, M. (2017). *ltmle: Longitudinal Targeted Maximum Likelihood Estimation*.

Seaman, S.R., Pavlou, M. and Copas, A.J. (2014). Review of methods for handling confounding by cluster and informative cluster size in clustered data. *Statistics in Medicine* **33**, 5371–5387.

Turner, E.L., Prague, M., Gallis, J.A., Li, F. and Murray, D.M. (2017). Review of recent methodological developments in group-randomized trials: Part 2-analysis. *Am J Public Health* **107**(7), 1078–1086.

van der Laan, M. and Rose, S. (2011). *Targeted Learning: Causal Inference for Observational and Experimental Data*. New York Dordrecht Heidelberg London: Springer.

van der Vaart, A.W. and Wellner, J.A. (1996). *Weak convergence and empirical processes*. Berlin Heidelberg New York: Springer.

Yang, G. (2021). Targeted learning for effect modification in randomized clinical trials and cluster randomized trials [Ph.D. Thesis]. University of Massachusetts, Amherst.

Zheng, W. and van der Laan, M. (2011). Cross-validated targeted minimum-loss-based estimation. In: van der Laan, M.J. and Rose, S. (editors), *Targeted Learning: Causal Inference for Observational and Experimental Data*. New York Dordrecht Heidelberg London: Springer.

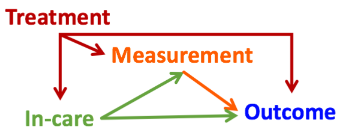

Fig. 1. Simplified causal graph to illustrate the challenges of adjustment for measurement impacted by the randomized treatment and post-baseline factors (here, being in care).
